# Syphilinum

**Published:** 1896-05

**Authors:** 


### SYPHILINUM.

Peevish, irritable, does not want to be touched or looked at.

Frets as long as a stranger is present.

Wants to be carried—can only be soothed by carrying up and down the room.

Crie cerebrale.

Unconsciousness.

Sensitive to noise.

Does not want to be left alone.

Wants a light in the room at night—fear in the dark.

Afraid of noises which he cannot account for.

Fear of mice.

Cries and thinks he will not get well.

Gloomy, despondent, taciturn.

Thought he was neglected by members of the family, which was not true.

Stupor—unconsciousness.

Rolling the head from side to side.

Bores the head into the pillows.

Scalp looks as though it had been dried on the skull.

Anterior fontanelle sunken in—has ceased to pulsate.

Sleeps with eyes half-open.

Eyes rolled back in head.

Sclerotics of both eyes red and congested, especially toward inner canthi (Hydrocephaloid).

Eyes dim, glazed; corneæ look as though they had been baked.

Little lumps of mucus on the eyeballs; eyeballs dry, no tears.

Ears very red, look as if the blood would burst out of them.

Face fiery red (during fever).

Face very much emaciated—old woman appearance (baby).

Profuse salivation.

Chewing motion of the mouth when asleep.

Tonsils inflamed and swollen, and fauces red and inflamed.

Deglutition very painful.

Yellowish gray, diphtheritic membrane on both tonsils.

Intense thirst for large quantities of water at frequent intervals.

Vomits water soon after drinking.

Vomits milk curdled.

Becomes pale about the mouth before she vomits.

Vomiting preceded by a hacking cough.

Vomits as if it were thrown out with a gush.

Gagging, retching, shows intense nausea and deathly sickness.

Very severe, cramping pain in the region of the umbilicus, ameliorated by heat, pressure, and sitting bent over. Rumbling in the abdomen constantly in the afternoon.

Stools watery, frequent (over twenty in twenty-four hours), yellow, and very offensive.

Sensation in rectum of two large extremely sore lumps which obstructed the passage, so that it was agonizing to have a stool.

Has to sit with legs wide apart so as not to press these two sore lumps together.

Desire for stool without result.

Not satisfied after stool, feels as if more ought to come.

Stools of hard, dry, brown lumps, excruciatingly painful—"would rather die than have a stool," though there is constant desire.

Pain in rectum worse from coughing, sneezing, blowing the nose, pressing to stool, standing and walking; only relief sitting with legs wide apart and heat (steam from hot water).

Red, sandy sediment in urine.

Urine very scanty, only twice in twenty-four hours.

"Root" of penis very much swollen and very sore.

Rich lemon-yellow, scanty urine (frequently confirmed).

Suppression of gonorrhœa by quack nostrums.

Prostate glands very much enlarged, inflamed, and very painful, as a result of suppressed gonorrhœa.

Hard, racking, tearing cough, which is restrained as much as possible on account of pain in the rectum.

Profuse expectoration of bright red blood, gushes of pure blood.

Pneumonia from suppressed gonorrhœa.

Swollen glands in nape of neck (Hodgkin's disease).

Automatic jerking of right arm and hand.

Four P. M. and two A. M. aggravations quite marked. Languid, taciturn, and indifferent during fever.

Perspiration, cold, clammy, smells musty, very profuse all over body and limbs during sleep.

Perspiration on the upper side of the body, while the side on which he lies is dry and hot.

Chilliness, even during high fever, worse from draft of air.

Dumb chill every other day about one P. M.

Chilliness up the back, worse when he moves.

Hands and feet cold, head hot.

Extreme emaciation, skin hangs in folds from limbs.

Epithelioma on neck, with sharp, darting, tearing pains in it; cicatrix blue, commenced to grow and enlarge after previous removal by cancer specialist; cured and remains cured to this date, three years after.

I omit from this summary the skin symptoms of the old lady, because they were not cured, but very much ameliorated.

The symptoms which I have enumerated were mostly intense and marked, were not affected by any previous medication, and were so quickly relieved and completely neutralized and removed that there could be no question that Syphilinum was the simillimum.

